# Assessment of Changes in Selected Features of Pine and Birch Wood after Impregnation with Graphene Oxide

**DOI:** 10.3390/ma17184464

**Published:** 2024-09-11

**Authors:** Izabela Betlej, Sławomir Borysiak, Katarzyna Rybak, Barbara Nasiłowska, Aneta Bombalska, Zygmunt Mierczyk, Karolina Lipska, Piotr Borysiuk, Bogusław Andres, Małgorzata Nowacka, Piotr Boruszewski

**Affiliations:** 1 Institute of Wood Sciences and Furniture, Department of Wood Science and Wood Protection, Warsaw University of Life Sciences—SGGW, 159 Nowoursynowska St., 02-776 Warsaw, Poland; izabela_betlej@sggw.edu.pl (I.B.); boguslaw_andres@sggw.edu.pl (B.A.); 2 Institute of Chemical Technology and Engineering, Faculty of Chemical Technology, Poznan University of Technology, 4 Berdychowo St., 60-965 Poznań, Poland; slawomir.borysiak@put.poznan.pl; 3 Department of Food Engineering and Process Management, Institute of Food Sciences, Warsaw University of Life Science—SGGW, 159 Nowoursynowska St., 02-776 Warsaw, Poland; katarzyna_rybak@sggw.edu.pl (K.R.); malgorzata_nowacka@sggw.edu.pl (M.N.); 4 Institute of Optoelectronics, Military University of Technology, gen. S. Kaliskiego 2 St., 00-908 Warsaw, Poland; barbara.nasilowska@wat.edu.pl (B.N.); aneta.bombalska@wat.edu.pl (A.B.); zygmunt.mierczyk@wat.edu.pl (Z.M.); 5 Institute of Wood Sciences and Furniture, Department of Technology and Entrepreneurship in Wood Industry, Warsaw University of Life Sciences—SGGW, 159 Nowoursynowska St., 02-776 Warsaw, Poland; karolina_lipska@sggw.edu.pl (K.L.); piotr_borysiuk@sggw.edu.pl (P.B.)

**Keywords:** graphene oxide, pine and birch wood, structural analysis, crystallinity, SEM, thermogravimetric measurements

## Abstract

In this work, pine and birch wood were modified by graphene oxide using a single vacuum impregnation method. The research results indicate that the impregnation of wood with graphene oxide increases the crystallinity of cellulose in both pine and birch wood, and the increase in crystallinity observed in the case of birch was more significant than in the case of pine. FT-IR analyses of pine samples impregnated with graphene oxide showed changes in intensity in the absorption bands of 400–600, 700–1500 cm^−1^, and 3200–3500 cm^−1^ and a peak separation of 1102 cm^−1^, which may indicate new C-O-C connections. In the case of birch, only some differences were noticed related to the vibrations of the OH group. The proposed modification also affects changes in the color of the wood surface, with earlywood containing more graphene oxide than latewood. Analysis of scanning electron microscope images revealed that graphene oxide adheres flat to the cell wall. Considering the differences in the anatomical structure of both wood species, the research showed a statistically significant difference in water absorption and retention of graphene oxide in wood cells. Graphene oxide does not block the flow of water in the wood, as evidenced by the absorbability of the working liquid at the level of 580–602 kg/m^3^, which corresponds to the value of pure water absorption by wood in the impregnation method using a single negative pressure. In this case, higher graphene oxide retention values were obtained for pine wood.

## 1. Introduction

Graphene and its derivatives can be used in many industries. Their unique properties mean they can be successfully used in pharmacy, medicine, electronics, energy, and environmental protection [1,2,3,4]. The latest research indicates that carbon nanomaterials can provide a wide range of applications in the wood technology industry and can be used to modify this natural material, giving it new unique properties [5,6]. Wood is an excellent utility material, but not without its drawbacks. It is flammable, susceptible to decomposition by fungi and termites, and hygroscopic. Modifying wood with nanomaterials such as graphene or graphene oxide improves and strengthens wood features significantly [7].

Graphene oxide (GO) is a material with a 2D structure composed of a single layer of carbon atoms attached to numerous oxygen functional groups that determine its properties. The presence of hydroxyl, epoxy, carboxyl, and carbonyl groups on the surface of graphene oxide makes it a reactive material that can modify and improve other materials’ properties [8]. Numerous literature reports suggest that graphene oxide may be effective for modifying wood and wood-based materials, improving their mechanical and physical properties, and influencing changes in flammability. Yan et al. [9] showed that graphene oxide in wood creates a physical and chemical barrier during the combustion process—it swells and emits gases, stopping the combustion reaction chain. In turn, Xu et al. [10] attributed the fire resistance of wood containing graphene oxide to pyrolysis—specifically, to a structure rich in C=C and C-O-C formed during pyrolysis.

In the works of other researchers, much attention was paid to strengthening the surface of natural fibers using graphene oxide, which resulted in improved mechanical properties and thermal stability of the modified fibers [11,12]. As Boudjellal et al. [13] indicated, such modified cellulose fibers have the potential for many advanced applications. Boudjellal et al. [14] propose solutions for the wood industry that have yet to be much considered. The study’s authors suggest that wood modified with graphene oxide can be used as sensors and heaters. Moreover, its surface is antistatic. Combinations of graphene oxide with polymers of natural origin have been recognized as a new group of hybrid materials whose unique properties will make them a new generation of materials.

Wood is a natural, renewable material used in various aspects of human life. Its unique anatomical structure makes the wood saturated with various chemically active ingredients. He et al. [15] showed that graphene oxide introduced into the wood capillaries lines the inner walls of cells. The same study authors suggested that the interaction between the nanomaterial flakes and the cell wall may have the character of electrostatic interactions. In turn, El Miri et al. [16] indicated the existence of interfacial compatibility and hydrogen bonds between GO and natural fibers.

In this research, two types of wood with different anatomical structures were modified by introducing graphene oxide into the cells using the single vacuum impregnation method. The research aimed to determine how graphene oxide influences changes in selected structural parameters and thermal stability of the material, which is wood, and whether the obtained differences between coniferous and hardwood are significant. Identifying these changes can considerably impact the purposeful and targeted modification of wood to obtain new properties not found in native wood. The most important research methods that allow us to indicate the interaction between wood and graphene oxide include X-ray diffraction and Fourier Transform Infrared Spectroscopy. These methods help conclude whether the interactions created are strong, whether new chemical bonds are formed, and how these changes will affect the basic parameters of wood, such as hygroscopicity. Comparative research on various types of wood also allows us to indicate which types or species of wood will be most susceptible for modification so they ultimately show the most significant potential for new applications. 

The aim of the undertaken research is of particular importance in the context of using wood as a universal material for many applications, especially in construction. Coniferous species are more often used as a technical material than deciduous species, which is associated with the greater availability of the former. Despite its multiple applications and excellent physical and mechanical properties, wood is stable only when not affected by variable humidity and temperature conditions. Wood is also a material that burns well. These factors mean that wood can be an unstable material. It can be subject to biocorrosion, which is associated with increased wood moisture, and the flammability of wood can cause substantial material losses and pose a severe threat. Therefore, utility wood, especially in construction, is permanently impregnated with hydrophobic preparations that protect the wood from moisture and, thus, from the development of biocorrosion. Based on the analyzed publications on the characteristics of graphene oxide, this material could be a component of impregnation formulations that prevent excessive wood hygroscopicity and reduce wood flammability. However, first, it is necessary to know how this material interacts with wood tissue so that appropriate modifications can be introduced and the mechanical properties of graphene oxide can be assessed. 

## 2. Materials and Methods

### 2.1. Preparation of Materials

**Figure 1:**
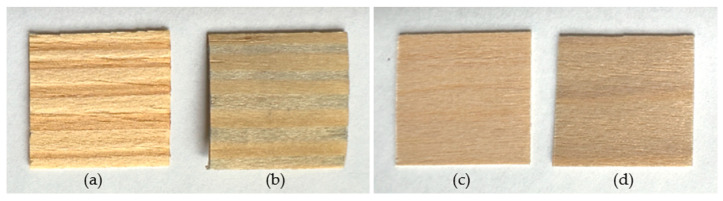
Samples: (**a**) control pine wood; (**b**) pine-GO; (**c**) control birch wood; (**d**) birch-GO.

The research material included pine samples (*Pinus sylvestris* L.) and birch (*Betula pendula* L.) with 25 mm × 25 mm × 0.4 mm dimensions. The sapwood was used for testing. Wood density at 12% humidity was 606 kg/m^3^ and 491 kg/m^3^ for pine and birch, respectively. An aqueous dispersion of graphene oxide (Advanced Graphene Products S.A., Zielona Góra, Poland) was used to impregnate wood, diluted with distilled water, obtaining a solution with a concentration of 0.08%. The diameter of the graphene oxide flake was 500–1000 nm. The suspension of graphene oxide in distilled water was stirred for 15 min using PS-40A ultrasound (CNC World Group Sp. z o. o., Jedlnia Letnisko, Poland) to reduce the amount of aggregating graphene oxide flakes in the solution. The samples were prepared for impregnation by placing them vertically in the solution, one above the other. They were loaded with glass balls to prevent the wood samples from floating to the surface. Impregnation of the samples was carried out in a vacuum dryer model 1445-2 (Sheldon Manufacturing Inc., Cornelius, OR, USA) with a pump—model V-700 (BUCHI Labortechnik AG, Flawi, Switzerland). The impregnation process was carried out under the following conditions: vacuum 190 mbar, time 30 min., temperature 28 °C. After this, the pressure in the autoclave was brought to atmospheric pressure, and the samples were left in the solution for another 30 min. The samples were then removed from the graphene oxide dispersion (Figure 1a–d). The retention of graphene oxide in the samples was determined based on the initial weight of the samples and their weight after impregnation.

### 2.2. Structural Analysis

#### 2.2.1. Microscopic Analysis

The visual presence of graphene oxide flakes in wood cells was confirmed using scanning electron microscopy (SEM) (Quanta 250 FEG SEM, FEI, Hillsboro, OR, USA). SEM images were acquired using an ETD-BSE backscattered detector (FEI, Hillsboro, OR, USA); the accelerating voltage was 10 kV, and the spot voltage was 3. Before imaging, the wood samples were coated with a 6.16 nm thick layer of gold. An EM ACE 600 sputtering machine (Leica Microsystems, Inc., Wetzlar, Germany) was used for this purpose. During the sputtering process, the table on which the samples were placed was rotated and tilted at an angle of 120°. 

Structural examinations of flat wood fragments were performed using a Zeiss SmartZoom 5 stereoscopic microscope (Carl Zeiss Meditec AG (Headquarter) Göschwitzer, Jena, Germany). A PalnApo D 1.6×/0.1 FWD 36 mm lens with LED lighting was used. During the examination of the samples, magnifications of 35× and 300× were used. At least three photos were taken for each research variant.

#### 2.2.2. FT-IR Analysis 

The infrared spectra were recorded by FT-IR (Fourier Transform Infrared Spectroscopy) on a Thermo Scientific spectrometer (Nicolet IS50, ThermoFisher SCIENTIFIC, Waltham, MA, USA). The presence of characteristic absorption bands was recorded. Samples were measured using the ATR technique (Attenuated Total Reflection) in a range of 400–4000 cm^−1^, with a resolution of 4 cm^−1^, and the number of scans was 64. The spectrum analysis was carried out on two flat wood surfaces. The spectra were analyzed using Omic software (ThermoFisher Scientific, Waltham, MA, USA). The tests were performed in duplicate.

#### 2.2.3. X-ray Diffraction Assay

X-ray diffraction (XRD) was measured using a SmartLab X-ray diffractometer (Rigaku Corporation, Tokyo, Japan). Analysis parameters: a Ni-filtered Cu Kα radiation source (1.5418 Å), voltage 40 kV, anode excitation 30 mA. The X-ray diffractogram was recorded for angles in the range of 2Θ = 5–30° with a step of 0.04°/3 s. The crystallinity (X_C_) of the materials was calculated as the ratio of the total area under the resolved crystalline peaks to the sum of the areas under the crystalline peaks and the amorphous curve. The following Formula (1) was used for calculations:

(1) $$X_{C}=\frac{\Sigma A_{C}}{{\Sigma A}_{C}+A_{A}}\%,$$

where:

$A_{C}$—total area under the resolved crystalline peaks, 

$A_{A}$—total area of amorphous phase.

#### 2.2.4. Thermogravimetric Measurements

The thermogravimetric measurements (TG) were carried out using a Netzsch TG 209F3 apparatus (Netzsch, Selb, Germany). The analyses were carried out in the temperature range of 40–700 °C at a heating rate of 10 K min^−1^ in a nitrogen atmosphere. 

### 2.3. Evaluation of Selected Physical Parameters

#### 2.3.1. Evaluation of the Contact Angle

The surface contact angle was determined using a Haas Phoenix 300 goniometer (Surface Electro Optics, Suwon City, Republic of Korea). An automatic droplet dispenser deposited a 1 μL water droplet on the wood surface. An image analysis system (Image XP, Surface Electro Optics, version 5.8, Suwon City, Republic of Korea) was used to determine the angle between the tangent to the droplet contour and the straight line intersecting its base. Measurements were taken at 5, 20, 40, and 60 s intervals from when the water droplet was deposited on the sample surface. Measurements were taken in the air with 50% humidity and a temperature of 21 ± 2 °C. Measurements were performed in 5 replicates.

The percentage contact angle change (W_Change n_) relative to start point of the measurements was calculated according to the following Formula (2):

(2) $$W_{Change\;n}=(({\mathrm{CA}}_{n}-{\mathrm{CA}}_{5})/{\mathrm{CA}}_{5})\times 100\%,$$

where:

W_Change n_—wettability change relative to initial value (contact angle measured in 5th second—CA_5_);

CA_n_—contact angle measured in n—second, n ∈ {5,20,40,60}.

#### 2.3.2. Assessment of Water Absorption

Water absorption measurements were carried out at time intervals after 10, 20, 30, 60, 120, and 240 min of immersing the veneers in water. Their complete immersion tested the water absorption of veneers in water. The water absorption was determined according to Formula (3):

(3) $$WA=((m_{1}-m_{2})/m_{1})\times 100\%,$$

where:

WA—water absorption in %;

m_1_—sample mass before immersion in water [g];

m_2_—sample mass after immersion in water [g].

Measurements were performed in 6 replicates.

### 2.4. Statistical Analysis

The results of graphene oxide retention in pine and birch wood were subjected to statistical analysis. One-way ANOVA was used for a significance level of α = 0.05. The statistical analysis of the results was carried out with the application Statistica version 13 (TIBCO Software Inc., Palo Alto, CA, USA).

## 3. Results and Discussion

The retention values of graphene oxide in pine and birch wood are presented in Table 1. Considering the differences in the anatomical structure of both types of wood, the tests revealed a statistically significant difference in water absorption and graphene oxide retention in wood cells (Table 1). Graphene oxide does not block the flow of water in wood, as evidenced by the absorption of the working liquid at the level of 580–602 kg/m^3^, corresponding to the absorption value of pure water by wood in the impregnation method using a single vacuum [17]. In the case of the tested types, higher graphene oxide retention values were obtained for coniferous wood. Based on the differences in retention, i.e., the amount of graphene oxide introduced into the wood, it is easier to conclude in which wood the interactions with graphene oxide are more robust, and which type of wood will be more beneficial for modification in terms of potential innovative applications. However, it should be noted that the percentage influence of wood species (7.8%) is much smaller than that of other factors not analyzed as part of the research—Error = 92.2% (Table 2).

### 3.1. Characterization of Structural Features

#### 3.1.1. Microscopic Examination Results

**Figure 2:**
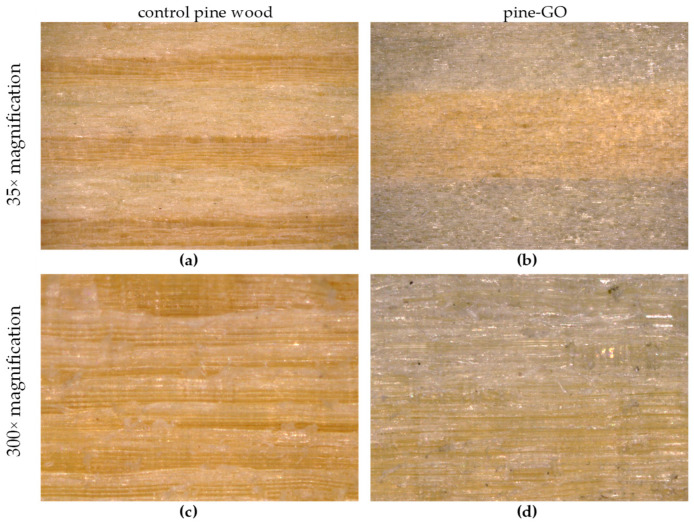
Visualization of the surface of pine wood samples before (**a**,**c**) and after (**b**,**d**) impregnation with graphene oxide, magnification: 35× (**a**,**b**) and 300× (**c**,**d**).

**Figure 3:**
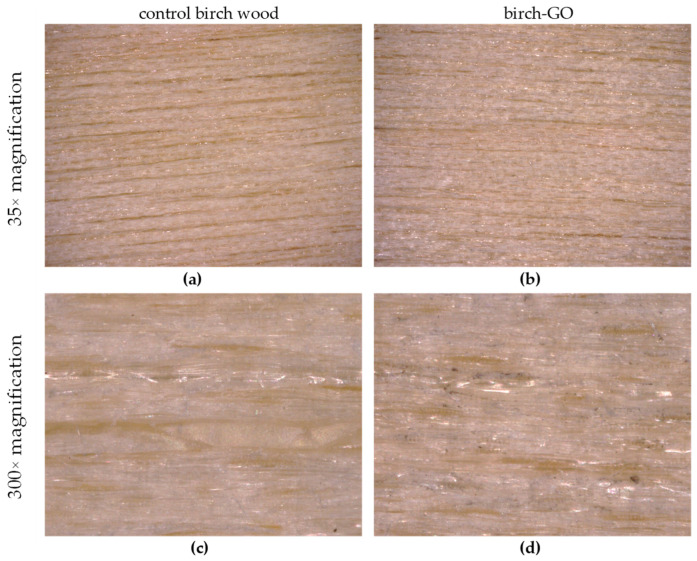
Visualization of the surface of birch wood samples before (**a**,**c**) and after (**b**,**d**) impregnation with graphene oxide, magnification: 35× (**a**,**b**) and 300× (**c**,**d**).

**Figure 4:**
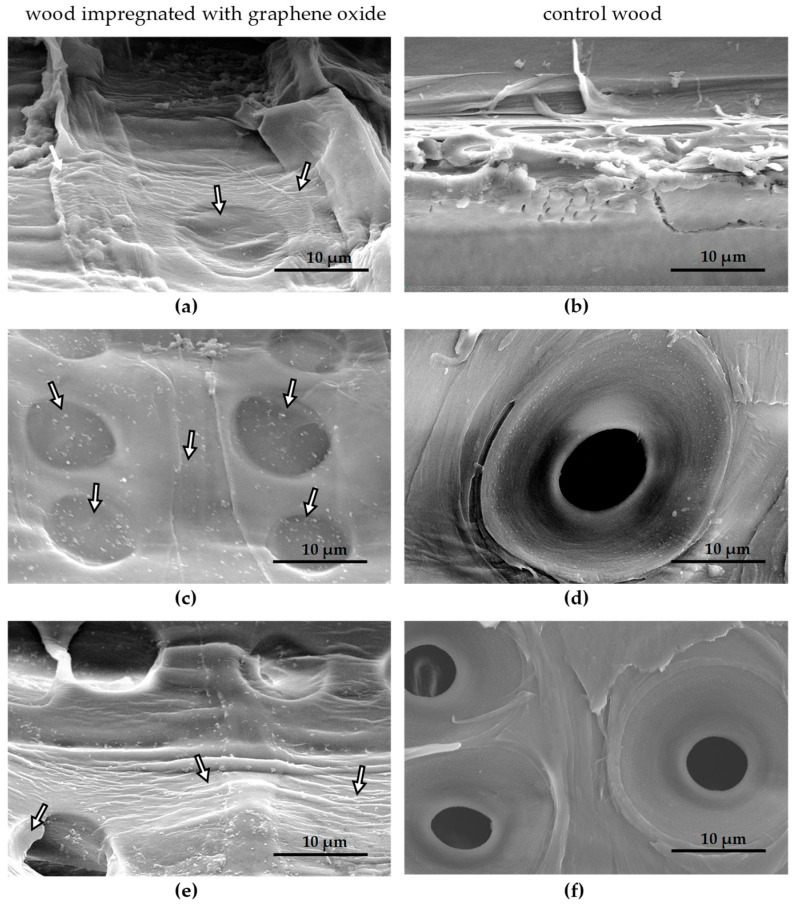
SEM microscope images of the interior of wood cells: (**a**) birch-GO; (**b**) control birch wood; (**c**,**e**) pine-GO; (**d**,**f**) control pine wood.

In order to identify morphological changes on the surface and in wood cells, microscopic examinations were carried out. In the optical microscope at 35× (Figure 2a,b and Figure 3a,b) and 300× (Figure 2c,d and Figure 3c,d), color changes of the wood surface are visible, related to the presence of the graphene oxide layer (Figure 2b,d and Figure 3b,d). It is visible that earlywood contains more graphene oxide than latewood (Figure 2 and Figure 3). This is related to better permeability of the anatomical cells that comprise this wood zone. SEM images at magnification 10,000× (Figure 4a–f) revealed the presence of graphene oxide (arrows) in wood cells of pine (Figure 4c,d) and birch (Figure 4a) in comparison to the control (Figure 4b—birch, Figure 4d,f—pine). Graphene oxide flakes adhere to the interior of the cell wall of conductive cells in wood, which is particularly visible in the places where funnel-shaped cavities are present (Figure 4a,c,e). In the SEM image, graphene oxide forms a semi-transparent and slightly wrinkled layer inside the wood cells. The arrows marked in the pictures indicate the places where the presence of a thin layer of graphene oxide flake is clearly visible. The interior of unimpregnated wood cells is characterized by a smooth surface of the cell wall and funnel-shaped cavity openings that are not clogged with anything (Figure 4b,d,e). The flat adhesion of graphene oxide flakes to the cell wall may be related to the interaction between GO and polymers contained in wood.

#### 3.1.2. FT-IR Analysis Results

**Figure 5:**
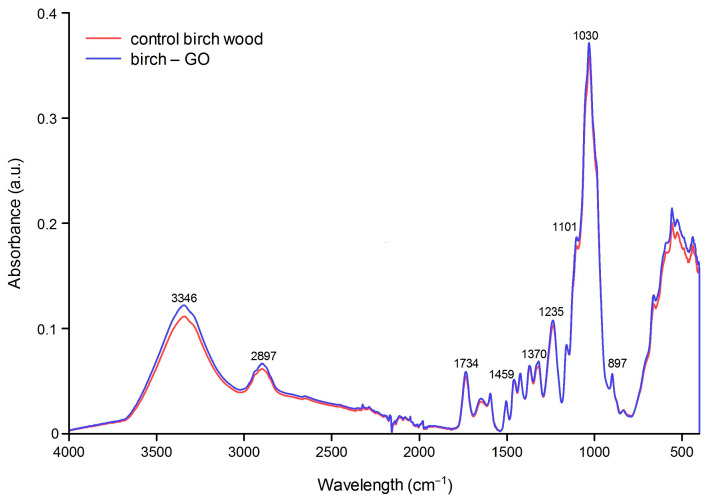
Characterization of FT-IR spectra of birch samples: control and impregnated with GO.

**Figure 6:**
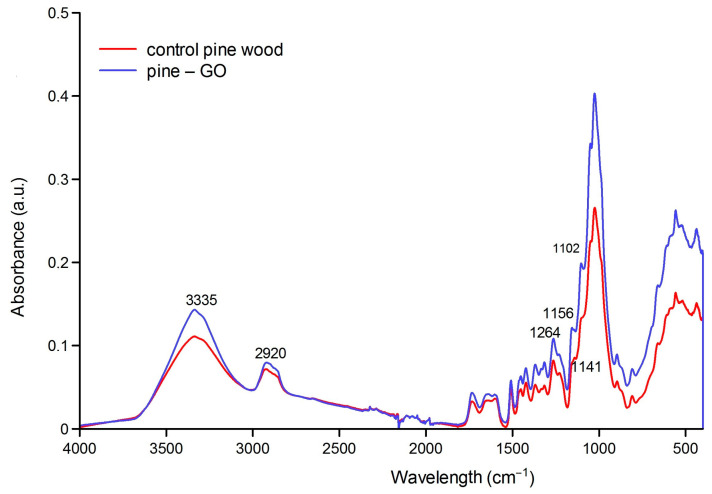
Characterization of FT-IR spectra of pine samples: control and impregnated with GO.

FT-IR images of pine and birch wood impregnated with graphene oxide are shown in Figure 5 and Figure 6. The 3600–2900 cm^−1^ area is characteristic of O-H and C-H stretching vibrations of polysaccharide chains. The broadband with a maximum of 3300 cm^−1^ corresponds to the hydroxyl group (-OH) stretching vibrations of polysaccharides. These will be both intra- and extramolecular bonds. The band in the area of 2800–2900 cm^−1^ is assigned to the -CH stretching vibrations in the carbon chain. In 1630–900 cm^−1^, cellulose has the following bands: 1633 cm^−1^ is the vibration of the water molecule absorbed by cellulose. Bands 1428, 1367, 1334, 1027, and 896 cm^−1^ are assigned to -CH_2_, -CH, -OH, and C-O bonds in cellulose. The band 1420–1430 cm^−1^ is associated with the degree of crystallization of cellulose, and the band 897 cm^−1^ with its amorphous form. Overall, higher peak intensities were recorded than in the analysis of control wood, and the most changed area was 950–1120 cm^−1^.

Birch samples do not show such noticeable changes (Figure 5). For this type of wood, areas 400–600 cm^−1^ and 3000–3500 cm^−1^ showed some differences, as connected with OH group vibrations. This slight increase may suggest the presence of GO. Visual assessment of the samples shows a lower GO content in wood structures, reflected in the FT-IR spectrum.

**Figure 7:**
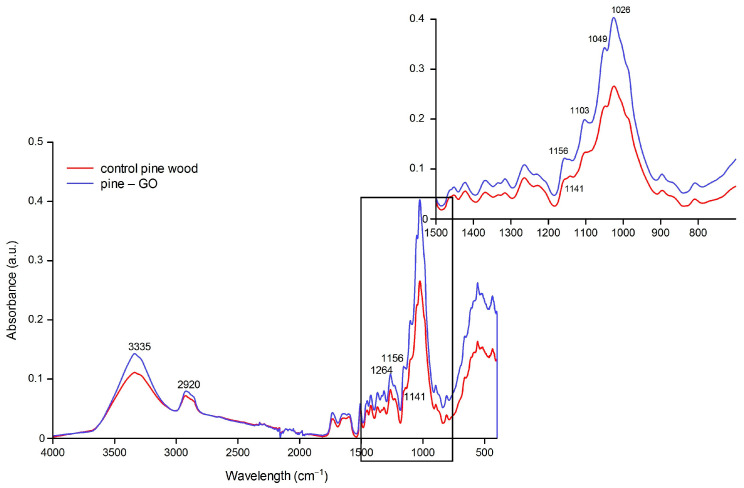
Pine samples spectrum with the magnification of the area in 1500–700 cm^−1^.

Pine samples showed different responses to GO impregnation (Figure 3). The changes were recorded in several absorption bands. The most effective ones were 400–600, 700–1500 cm^−1^, and 3200–3500 cm^−1^. In the second area, one can observe the separation of the 1102 cm^−1^ peak, which may indicate new C-O-C connections between GO and wood. Also, a shift in the 1112–1160 cm^−1^ area can be observed. Figure 7 shows the peak shift from 1114 to 1156 cm^−1^ connected with a stretching C-O-C bridge. An increase in peak intensity was observed in the broad band of 3500–3200 cm^−1^ corresponding to the vibrations of -OH bonds, which may contribute to an increase in the hygroscopicity of wood [6,18]. The increase in the intensity of the spectrum in the wavelength range of 3500–3200 cm^−1^ may result from the increase in the number of -OH groups resulting from the presence of graphene oxide. As suggested by Wang et al. [19], an increase in the intensity of the peak in the band attributed to -OH stretching vibrations may also indicate the binding of GO to the wood cell wall through hydrogen bonds. Other researchers also confirm this phenomenon [20].

#### 3.1.3. XRD Analysis and the Degree of Crystallinity

**Figure 8:**
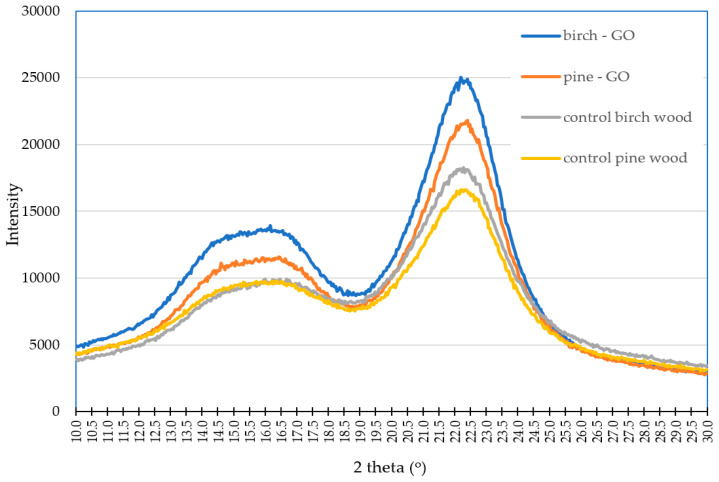
XRD patterns of wood impregnated with graphene oxide.

XRD analysis characterizes changes in the cellulose structure of pine and birch wood due to impregnation with graphene oxide (Figure 8). The profile of the presented curves is characteristic of cellulose, and the positions of the maxima at angles 2θ indicate the presence of polymorphic cellulose I [21]. Three characteristic peaks have been distinguished, which correspond to lattice planes with Miller indices of (010), (110), and (200). These peaks were characterized at angles of 14.92° (pine wood), 14.96° (birch wood), 16.32° (birch wood), 16.36° (pine wood), and 22.48° (pine and birch wood) (Figure 5). The diffractometer showed an apparent increase in the peak intensity at 2θ = 22.48° in wood impregnated with graphene oxide. Additionally, the peak for birch wood was more extensive than pine wood. These changes may be caused by defects in the crystal structure, which is related to the interaction of graphene oxide with cellulose. The diffraction patterns of treated wood are consistent with those without graphene oxide. There is no characteristic peak for graphene oxide, which is observed at 2θ = 11.4, as suggested by Li et al. [22], which may be related to the disappearance of oxygen-containing groups in graphene oxide, which form chemical bonds with polymer groups of the wood cell wall. Regarding the results of FTIR tests, it should be ruled out that the increase in the intensity of the diffraction peak of the GO-impregnated birch sample is related to the formation of new bonds between wood polymers and graphene oxide. Perhaps other interactions should be considered, such as amorphous scattering [21] or physical interactions [23,24].

The impregnation of wood with graphene oxide increases the crystallinity of cellulose in pine and birch wood (Table 3). However, the increase in crystallinity observed for birch was more significant than for pine. A similar increase in cellulose crystallinity in the presence of GO was found by Gabryś and Ślusarczyk [25], simultaneously indicating a correlation between the increase in cellulose crystallinity and the concentration of graphene oxide. The authors of the publication found that at high GO concentrations, the increase in cellulose crystallinity begins to slow down. Kumar et al. [26] suggest that the increase in cellulose crystallinity is conditioned by numerous hydrogen bonds resulting from new connections between cellulose and graphene oxide. 

Considering the changes occurring in wood under the influence of GO and trying to explain these changes in the context of pine and birch wood is not an easy matter. Wood is a very complex material in terms of anatomy (different anatomical structures between species and a completely different anatomical structure between deciduous and coniferous trees) and chemistry. Wood is also a material composed of three polymers with a complicated chemical composition (especially lignin), which shows a different degree of polymerization in different species. Coniferous species are characterized by a higher cellulose and lignin content in the cell wall than deciduous wood species [27]. The latter, in turn, is characterized by the presence of hemicelluloses rich in pentosans, which in coniferous wood constitute a small percentage [28]. The differences in crystallinity between pine-GO and birch-GO should be considered in many aspects. The wood species should be considered, as well as its anatomical structure and the chemical composition of the cell wall, which are the cell structures in which graphene oxide flakes are located. Ling et al. [29] indicated that chemical and physicochemical modification of wood causes changes in the degree of cellulose crystallinity due to the reduction of amorphous sites in cellulose by some chemical components. Other researchers indicate that during chemical modification, changes in the content of amorphous xylan and some lignin fractions may occur, which may also lead to an increase in the degree of biomass crystallinity [30].

#### 3.1.4. TG Study of Wood Samples

**Figure 9:**
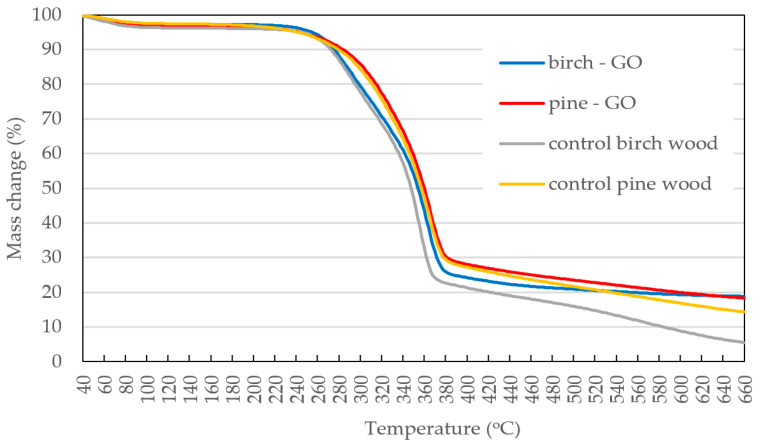
TG thermographs of wood samples impregnated with graphene oxide.

Thermogravimetric curves of impregnated and control wood are shown in Figure 9. The initial and similar weight loss of treated and impregnated wood was related to the evaporation of free water and volatile substances in wood cells. The second mass loss observed in the temperature range from 270 °C to 380 °C corresponds to the decomposition of hemicelluloses and then cellulose. Also, the differences between control and impregnated wood are slight in this respect. Some differences are observed between wood species, related to the difference in the share of polysaccharides in the cell wall. In the temperature range from 360 °C to 660 °C, the increased thermal stability of impregnated wood was observed, especially birch wood. The weight loss practically did not change. Publications by other researchers also indicate that GO improves the thermal stability of wood [31]. Baishya and Maji [32] showed that the residual mass content of cellulose-GO composite increases with the increase of graphene oxide in the composite. Oluwasina et al. [33] explain the more excellent thermal stability of the wood-GO composite primarily by the higher carbon to oxygen ratio compared to native wood. However, Boudjellal et al. [34] see the increased thermal stability as being due to strong interactions between cellulose and graphene oxide.

In the studies of other authors dealing with the assessment of the effect of GO on the quality of polymer composites, it was found that the increase in thermal stability results from interphase interactions through the formation of hydrogen bonds and van der Waals forces [35]. Ye et al. [36] observed an increase in the decomposition temperature of wood in a WPC composite with the addition of GO, and the cause of this phenomenon was seen in the presence of non-agglomerated GO, which creates a barrier delaying the release and decomposition of volatile products. Similarly, other 2D carbon materials, such as carbon nanotubes or graphene, a component of polymer composites containing wood particles, increase thermal stability [37]. 

The presented studies found that GO increases the thermal stability of birch wood, which was not observed in the case of pine wood. The differences that occurred should be sought in the anatomical structure and chemical composition of polymers that are components of the cell walls of conductive capillaries in which GO is oxidized. In FT-IR analyses, it was found that chemical changes occur in pine wood modified with graphene oxide, which was not observed in the case of birch-GO. Therefore, chemically unbound GO may lead to agglomeration of high char residues, which cannot be further separated into smaller volatile fragments.

### 3.2. Characteristics of Physical Parameters

#### 3.2.1. Contact Angle Results

**Figure 10:**
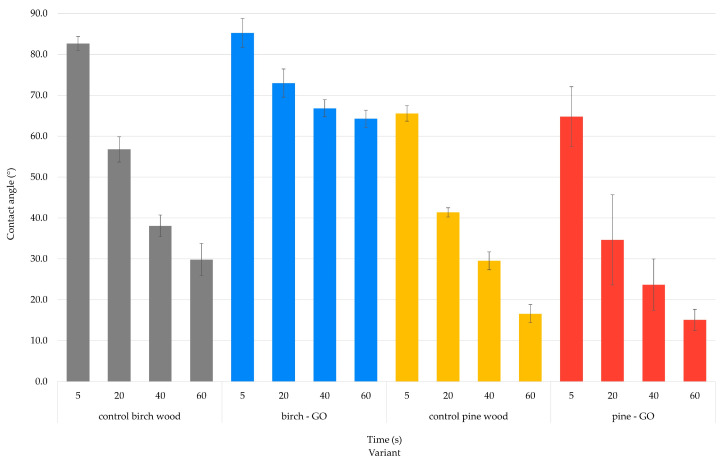
Wet angle changes after 5, 20, 40 and 60 s.

**Figure 11:**
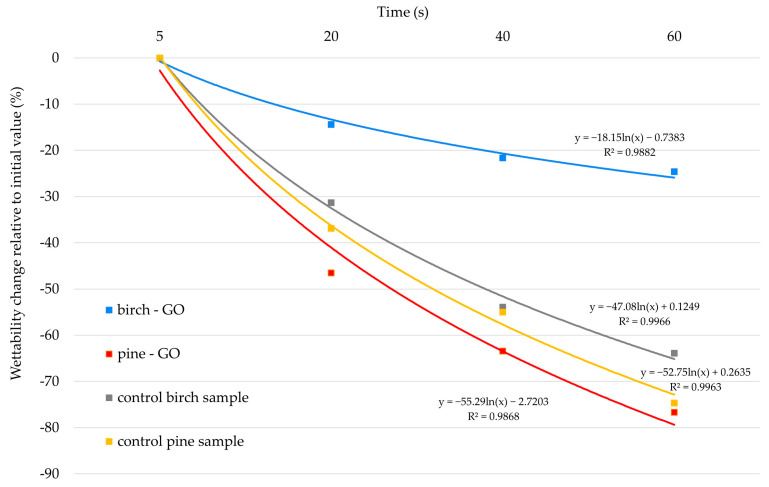
Percentage change in wetting angle over time for GO-treated and control wood.

The results of the wetting angle change over 5, 20, 40, and 60 s during the water wettability tests are presented in Figure 10. Additionally, Figure 11 shows the percentage change in the wetting angle relative to the starting point of the measurements.

The wettability of control wood samples was similar for birch and pine, with pine showing higher hygroscopicity than birch wood. Impregnation of birch with graphene oxide changed its properties towards hydrophobicity, which was not confirmed in pine impregnated with the same material. The percentage change in wettability of birch-GO samples was less than 25%. In contrast, in control samples, it was 64% (Figure 11), which indicates the influence of graphene oxide on changes in the wettability of the surface of this wood species. The percentage change in wettability of pine wood surface impregnated with GO was 2% higher than in wettability observed in control pine.

Figure 11 shows changes in wood wettability in percentage terms. As can be easily seen, the percentage of changes in the birch-GO wetting angle after 60 s from applying a water droplet to the surface was the smallest of all the variants tested (it was approx. 25%). GO gives the birch wood surface a much less hygroscopic (more hydrophilic) character than the control wood surface. The percentage change in the pine-GO surface wettability was less than 80% and did not statistically differ from the changes observed on the control wood surface.

Analyzing various factors influencing the surface wetting angle (Table 4), it should be noted that wood species, graphene oxide impregnation, time from drop deposition, and interactions between these factors had a statistically significant effect on the obtained angle values (*p* < 0.05). Among the main factors, the highest percentage effect on the wetting angle values was demonstrated by wood species (X = 32.7%) and time from drop deposition (X = 49.9%). A percentage effect of 2.9% characterized graphene oxide impregnation. At the same time, it should be noted that the interaction between wood species and graphene oxide content was characterized by a more than twice as high percentage effect (X = 7.7%). This indicates a different effect of graphene oxide impregnation on individual wood species. It is also worth noting that the effect of factors not analyzed in this study was only Error = 1.8%.

Previous studies conducted by the co-authors show that materials with a GO layer on the surface exhibit hydrophobic properties [38]. Literature data indicate that GO layers are currently tested as potential anti-corrosion layers [39]. Studies conducted on birch-GO confirm these relationships. Similar results were not obtained for pine wood. The cause of this phenomenon can be found in the anatomical structure of wood. This assumption is confirmed by the results of microscopic studies, which indicate differences in the color of pine and birch wood surfaces after impregnation with graphene oxide. The lack of color change in the late pine wood zone indicates that the cells in this zone were less saturated with GO, which may translate into more minor differences in surface wettability. Łukawski et al. [37] suggest that the wood species and the method of applying the tested substance determine the changes in surface wettability. The study’s authors indicated that superhydrophobic properties can be obtained by using the drop-casting or dip-coating method. Graphene surfaces’ wettability increases when GO is evenly distributed on the surface [40]. In the case of graphene oxide dispersion in water, the flakes can form a “rose petal” structure on the surface, which occurs as a result of friction of the flakes, and this can promote a decrease in the wetting angle [41]. It seems, therefore, that the applied impregnation method is more helpful in modifying the properties of birch wood than pine. 

#### 3.2.2. Water Absorption Results

**Figure 12:**
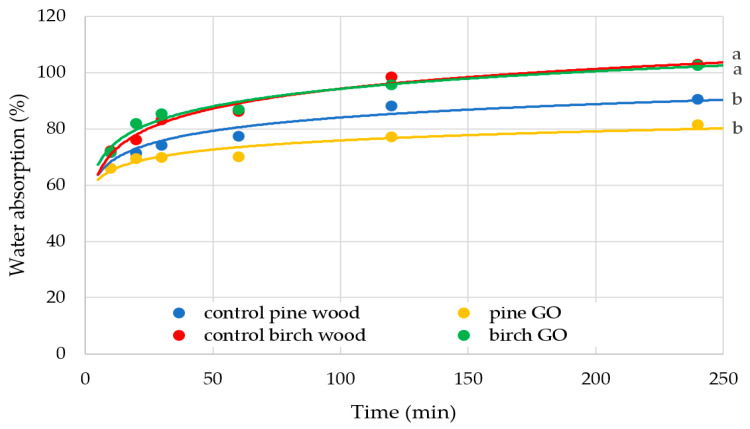
Water absorption by wood samples; a, b—homogeneous groups by the Tukey test.

The impregnation of veneers with graphene oxide does not affect their water absorption (Figure 12). Only the wood species (different homogeneous groups: a, b) showed a statistically significant effect (*p* < 0.05). The percentage effect of wood species on the water absorption value was 52.4% (Table 5). Neither impregnation with graphene oxide nor the interaction between graphene oxide content and wood species showed a statistically significant effect on the water absorption values of veneers (*p* > 0.05). This is the opposite relationship from that observed in the case of the surface wettability test. This indicates a limited effect of graphene in the case of intensive moisture exposure—the samples were wholly immersed in water. It is worth noting that the water absorption of veneers is greatly influenced by other factors not analyzed during these tests (Error = 39.7%).

The wettability and water absorption of wood are some of the most important properties that determine the direction of its applications. Wood is a hygroscopic material; therefore, it is often subjected to chemical and physical modification to reduce water sorption. Scientific publications indicate that graphene oxide changes the wettability of materials in the hydrophobic direction [42,43]. However, the deposition method of graphene oxide on the surface of materials is of great importance here [23]. Graphene oxide itself is a hygroscopic material, but the way of absorbing water molecules depends on the structure of graphene oxide and external conditions. Riza et al. [44] state that the relative humidity of the air in the environment affects how graphene oxide interacts with water. In turn, Lian et al. [45] explained that micrometer-sized tunnel-like wrinkles on the laminated GO surface affect the capillary pressure and, thus, the water absorption capacity.

In the presented studies, no statistically significant differences were observed between the control and the modified sample. However, differences in water absorption between wood species are visible, which result from differences in anatomical structure. 

## 4. Conclusions

In this study, we impregnated wood with graphene oxide using low-pressure methods. The study aimed to verify the interaction of graphene oxide with hardwood and coniferous wood. The statistical analysis of the retention of graphene oxide in wood cells showed that the anatomical structure of the wood has a minor impact on the differences in the obtained retention compared to other undefined factors. The analysis of structural tests showed that graphene oxide contributes to the increase in the crystallinity of cellulose. In the case of pine and birch wood, this increase may result from different interactions between GO and cellulose. In the case of pine wood, chemical interactions and the formation of new bonds are visible. In the case of birch wood, the increase in crystallinity may result from other interactions not specified in the publication. The flat adhesion of graphene oxide flakes to the cell wall surface, observed in studies using SEM microscopy, indicates interactions of a physical nature. TG analysis indicates that adding graphene oxide reduces the complete combustion of wood. Despite lower retention of graphene oxide in birch wood, this species showed more excellent thermal stability in the temperature range from 380 °C to 660 °C, which may indicate that the method of interaction between GO and birch wood is different from that observed in pine wood.

Based on wettability studies, graphene oxide affects changes in wood wettability, but these are strongly related to wood species. Therefore, a specific selectivity can be observed. Not every wood species will be suitable for modification with graphene oxide.

The obtained results may significantly impact further planned experiments and research hypotheses. Searching for ways to modify wood is essential to maintain its durability in various, often demanding, use environments, and to acquire new properties. A detailed understanding of the mechanisms of interaction between GO and wood may help in making appropriate and goal-oriented decisions regarding the use of this substance in the wood industry. 

Based on the results, not every wood species will be suitable for modification with graphene oxide. The complex anatomical structure, different in all species, may be a factor limiting the common use of GO. Nevertheless, graphene oxide may have great potential in wood technology.

Graphene oxide introduced into wood may modify its structural features, impacting its most important functional properties. The increased cellulose crystallinity in wood modified with graphene oxide may result in better mechanical properties. The physical properties of wood are also significantly improved. The change in the wetting angle observed in birch wood modified with GO may result in lower moisture absorption on the wood surface, and this feature is essential from the point of view of wood protection against abiotic and biological corrosion. 

However, in order to fully confirm that graphene oxide can be considered a good modifier of wood properties, several additional tests should be carried out, including the assessment of strength parameters and analysis of other features necessary for materials engineering, such as flammability or protection against fungi and termites.

## Figures and Tables

**Table 1:** Retention of graphene oxide in wood.

Samples	Solution Retention		Graphene Oxide Retention	
	Average Content	SD	Average Content	SD
(kg/m^3^)				
pine-GO	602 ^a^	35.17	48.16 ^a^	2.81
birch-GO	580 ^b^	40.01	46.40 ^b^	3.71

SD—standard deviation; ^a,b^ are homogeneous groups by the Tukey test.

**Table 2:** Analysis of variance in terms of the significance of the impact of kinds of wood.

	*p*-Value	X
kind of wood	0.001962	7.8
Error		92.2

*p*—probability of error, X—percentage (%) influence of kinds of wood.

**Table 3:** Changes in cellulose crystallinity in wood impregnated with graphene oxide.

Samples	Degree of Crystallinity X_c_ (%)
control pine wood	53
pine-GO	61
control birch wood	56
birch-GO	66

**Table 4:** Variance analysis of the influence of factors on the contact angle results.

Factor	*p*-Value	X
kind of wood	<1.00 × 10^−17^	32.7
GO content	4.14 × 10^−8^	2.9
time from application of drops	<1.00 × 10^−17^	49.9
kind of wood × GO content	5.10 × 10^−13^	7.7
kind of wood × time from application of drops	1.89 × 10^−3^	1.1
GO content × time from application of drops	2.72 × 10^−5^	2.0
kind of wood × GO content × time from application of drops	2.14 × 10^−5^	2.0
Error		1.8

*p*—probability of error, X—percentage (%) influence of factors.

**Table 5:** Variance analysis of the influence of factors on the water absorption results.

Factor	*p*-Value	X
kind of wood	0.000001	52.4
GO content	0.095438	4.2
kind of wood × GO content	0.118515	3.7
Error		39.7

*p*—probability of error, X—percentage (%) influence of factors.

## Data Availability

The original contributions presented in the study are included in the article, further inquiries can be directed to the corresponding author.
